# The immune and microbial homeostasis determines the *Candida*–mast cells cross-talk in celiac disease

**DOI:** 10.26508/lsa.202302441

**Published:** 2024-05-08

**Authors:** Giorgia Renga, Marilena Pariano, Fiorella D’Onofrio, Giuseppe Pieraccini, Claudia Di Serio, Valeria Rachela Villella, Carlo Abbate, Matteo Puccetti, Stefano Giovagnoli, Claudia Stincardini, Marina Maria Bellet, Maurizio Ricci, Claudio Costantini, Vasileios Oikonomou, Luigina Romani

**Affiliations:** 1 Department of Medicine and Surgery, University of Perugia , Perugia, Italy; 2 Mass Spectrometry Centre (CISM), University of Florence, Florence, Italy; 3 Department of Experimental and Clinical Medicine, University of Florence, Florence, Italy; 4 European Institute for Research in Cystic Fibrosis (IERFC-Onlus), San Raffaele Scientific Institute, Milan, Italy; 5 Department of Pharmaceutical Sciences, University of Perugia , Perugia, Italy

## Abstract

Celiac disease (CD) is an autoimmune enteropathy resulting from an interaction between diet, genome, and immunity. Although many patients respond to a gluten-free diet, in a substantive number of individuals, the intestinal injury persists. Thus, other factors might amplify the ongoing inflammation. *Candida albicans* is a commensal fungus that is well adapted to the intestinal life. However, specific conditions increase *Candida* pathogenicity. The hypothesis that *Candida* may be a trigger in CD has been proposed after the observation of similarity between a fungal wall component and two CD-related gliadin T-cell epitopes. However, despite being implicated in intestinal disorders, *Candida* may also protect against immune pathologies highlighting a more intriguing role in the gut. Herein, we postulated that a state of chronic inflammation associated with microbial dysbiosis and leaky gut are favorable conditions that promote *C. albicans* pathogenicity eventually contributing to CD pathology via a mast cells (MC)-IL-9 axis. However, the restoration of immune and microbial homeostasis promotes a beneficial *C. albicans*–MC cross-talk favoring the attenuation of CD pathology to alleviate CD pathology and symptoms.

## Abstract (precis)

This study shows that the restoration of immune and microbial homeostasis promotes a beneficial *Candida albicans*–MC cross-talk attenuating the intestinal pathology in celiac disease.

## Graphical Abstract

**Figure:**
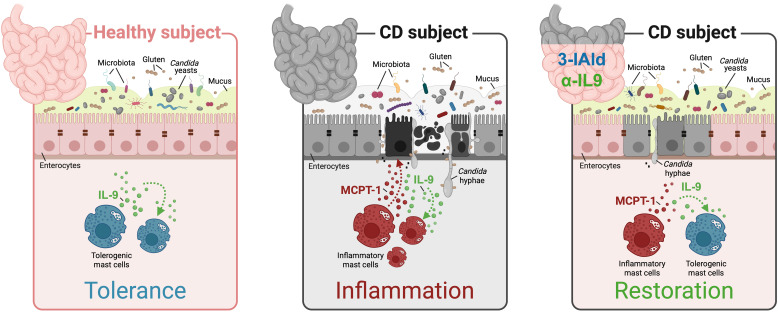

## Introduction

Celiac disease (CD) is a chronic immune-mediated enteropathy that primarily affects the small intestine and occurs from aberrant cellular responses to gluten peptides in genetically susceptible individuals expressing the human leukocyte antigen (HLA) DQ2 or DQ8 (1). The clinical spectrum of CD includes classic gastrointestinal symptoms but also extra-intestinal manifestations of cutaneous, endocrinological, and neuropsychiatric nature (2). Like in many autoimmune diseases, antibody production against gliadin, tissue transglutaminase 2 (TG2), and endomysium is an important feature in CD and indicates a loss of immune tolerance to self-antigens (3). Being a multifactorial disease, the complementary genetic and environmental factors mainly influence CD penetrance and are likely necessary to break intestinal tolerance to gluten, thus promoting intestinal tissue damage. However, only a small fraction of high-risk individuals exposed to gluten develop CD, indicating that additional environmental factors are likely to have a role in the development of disease (1). Mounting evidence implies that several infectious agents, such as viruses (4, 5), or other yet-to-be-defined factors may act as triggers in CD (6).

In 2003, reference 7 have hypothesized that *Candida albicans*, a human commensal of the gastrointestinal and genital tracts (8), may be a triggering factor in CD after the observation of similarity between the fungal wall component, hyphal wall protein 1 (Hwp1), and two CD-related gliadin T-cell epitopes (7). This hypothesis has been supported by the observation of higher levels of anti-Hwp1, anti-gliadin and anti-TG2 antibodies in the serum of CD patients more than healthy controls and by the detection of *Candida* sp. in 33% of CD fecal specimens compared with 0% of the control group (9, 10). As a successful colonizer of the human host, *C. albicans* has the ability to survive by adapting to a wide range of host niches through its metabolic and morphological flexibility. The fungus can take on the most common morphologies of yeasts, generally associated with commensalism, and hyphae, considered the most invasive form (11). The switch from harmless colonizer to virulent pathogen is, in most cases, due to perturbation of the fungus–host microbiota interplay and is finely regulated by inflammation (12). A homeostatic microbiome exerts an inhibitory effect on *Candida* adhesion, colonization and invasion. On the contrary, gut dysbiosis promotes *C. albicans* colonization. Being a gastrointestinal inflammatory disease with a dysbiotic print and leaky gut (13, 14), CD seems to have favorable conditions for the commensal-to-pathogen switch of *C. albicans*.

Inflammation is a key player in CD. It has been proposed that there is an inflammatory preclinical set up preceding the disease on which various environmental inflammatory stimuli can insist. This constitutive alteration can predispose to autoimmune condition in which gluten exacerbates inflammation (15). In this scenario, mast cells (MCs) may represent important drivers of intestinal inflammation. MCs are tissue-resident cells typically located at the strategical sites and involved in host defense. MCs can either regulate homeostasis or promote inflammatory processes through the release of specific mediators (16). Several reports have documented an increased MC number in the untreated CD subjects that returns to normal levels after gluten withdrawal (17). In addition, Frossi et al have found that the increased density of infiltrating MCs in CD intestinal biopsies correlates with an increased inflammatory grade, according to the Marsh classification, and MCs were found to directly respond to non-immunodominant gliadin fragments by releasing a large amount of inflammatory cytokines in vitro (18). These observations indicate that MC plasticity may be a double-edged sword in CD and as-yet uncharacterized environmental changes can drive MCs to exacerbate mucosal inflammation. Previously, we demonstrated that MCs work together with IL-9, the key cytokine that autocrinally drives mastocytosis (19), in promoting barrier function loss and fungal dissemination. Thus, the IL-9–MC axis could act as a signature that discriminates between the pathogenic versus protective role of *C. albicans* in the gut (20).

In the present study, we postulated that a state of chronic inflammation associated with microbial dysbiosis and impaired intestinal barrier function are favorable conditions that promote *C. albicans* pathogenicity eventually contributing to CD pathology. By resorting to in vitro and in vivo preclinical models of CD, we have found that the *C. albicans*–MC cross-talk is contingent upon the underlying inflammatory state and dysbiosis. However, the restoration of immune and microbial homeostasis promotes a beneficial *C. albicans*–MC cross-talk and attenuates the intestinal pathology in CD. Thus, a better understanding of the *Candida*–host microbiota cross-talk provides important advances in the pathogenesis and therapy of human CD.

## Results

### Gliadin peptides affect *C. albicans* morphotype

**Figure S1.**
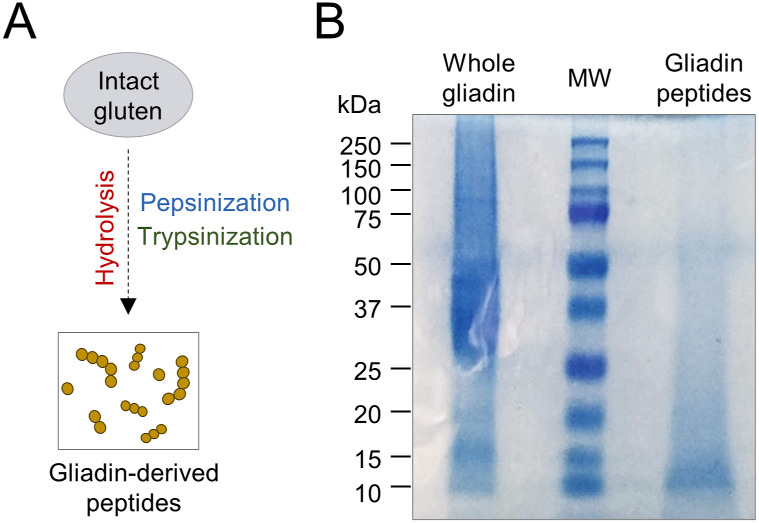
Electrophoretic characterization of gliadin peptides after enzymatic digestion. **(A, B)** Hydrolysis phases’ schematic description and (B) gel electrophoresis of whole gliadin-digested peptides, stained with Coomassie brilliant blue. MW, molecular weight (kD).

**Figure 1.**
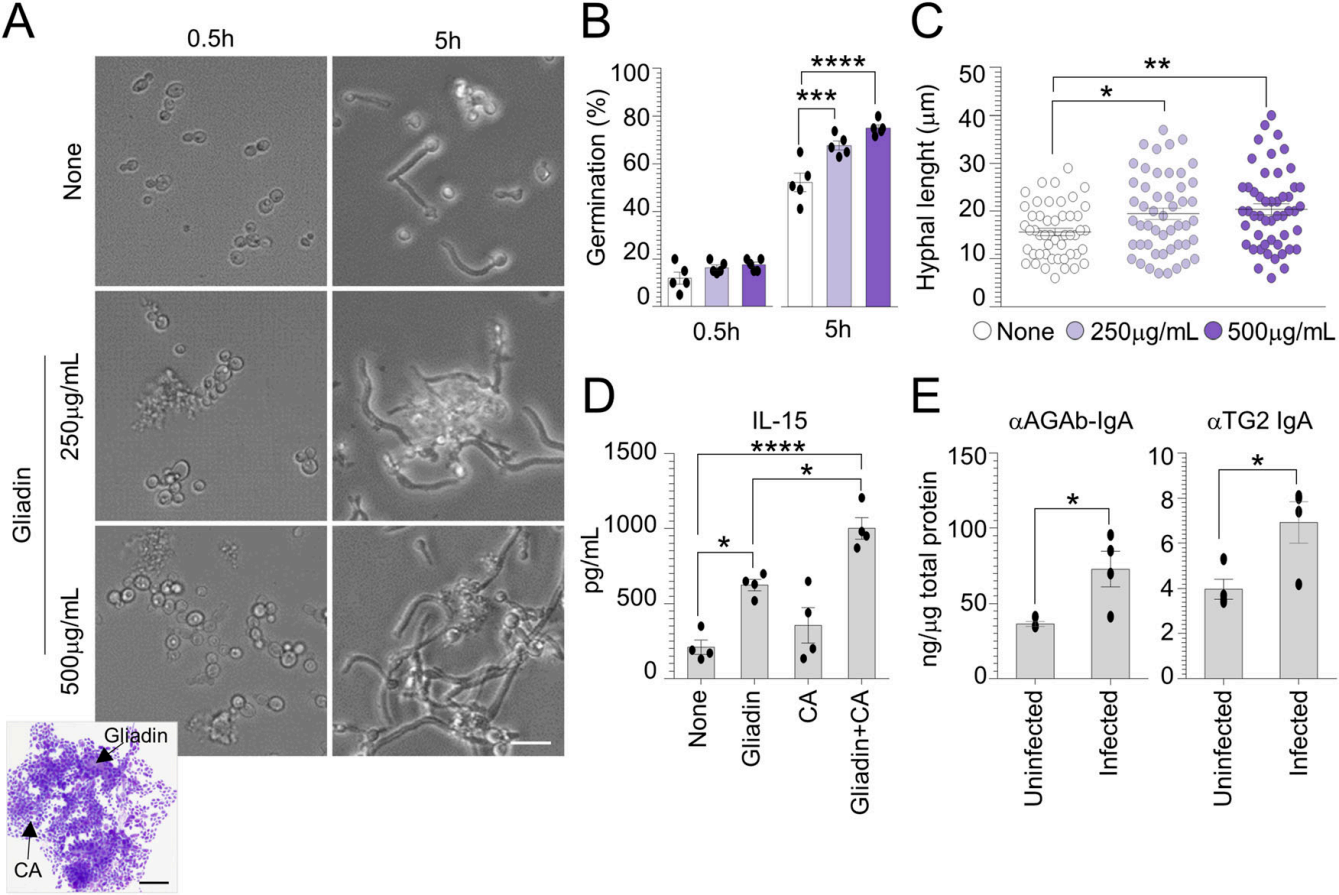
*Candida albicans* contributes to celiac disease initiation and development. *Candida albicans* (SC5314) yeasts were exposed at different concentrations of pepsin–trypsin–gliadin peptides in RPMI-1640 liquid medium. **(A, B, C)** Cells aliquots were imaged (A) by microscopy and May–Grünwald Giemsa staining (on inset) and evaluated for (B) cell germination and (C) hyphal length (a minimum of 50 hyphae were analyzed in different high-power fields). Images were taken with a high-resolution microscope (EVOS FL Auto Cell Imaging System), 100× magnification (scale bar, 20 μm). **(D)** Caco-2 cells were pulsed with *C. albicans* and treated with 500 μg/ml gliadin peptides for 5 h and evaluated for (D) IL-15 production by ELISA. **(E)** WT mice fed with standard diet were infected intragastrically with 1 × 10^9^ *Candida albicans* and evaluated for (E) anti(α)-gliadin (AGAb) and α-TG2 IgA production by ELISA in the small intestine. Data are expressed as mean ± SEM of two independent experiments. In (E), n = 4 mice/group. *P < 0.05, **P < 0.01, ***P < 0.001, ****P < 0.0001. One-way ANOVA, two-way ANOVA, or unpaired *T* test, Tukey’s multiple comparisons test. Treated versus untreated cells (None), gliadin- versus gliadin+CA–treated cells, infected versus uninfected mice. CA, *Candida albicans*. Source data are available for this figure.

To test a potential interaction between gliadin peptides and *C. albicans*, we first evaluated the effects of gliadin peptides on *C. albicans* growth and germination. To this purpose, commercial wheat gliadin was subjected to enzymatic peptic–tryptic digestion (Fig S1A and B) and different concentrations of pepsin–trypsin resistant (PT)-gliadin peptides were added to *C. albicans* cultured at 37°C for 5 h in an amino acid–rich liquid medium (RPMI-1640). We found that PT-gliadin peptides were able to promote the formation of large cell–cell aggregates at 0.5 h and germination at 5 h (Fig 1A and B). In addition, gliadin-treated *Candida* cells displayed a higher hyphal length when compared with untreated cells (Fig 1C). Previous observations have demonstrated that hyper-virulent strains of *C. albicans* have highly aggregative colony structure in vitro and more extensive cell damaging activity within the oral epithelial layers (21). Thus, the ability of gliadin peptides to promote aggregation and increase hyphal growth could reflect a higher capacity of *Candida* to adhere and damage epithelial cells and trigger an inflammatory response. To test this hypothesis, proliferating Caco-2 cells were exposed to *C. albicans* treated with gliadin peptides for 5 h and evaluated for the levels of IL-15, a critical player in CD pathogenesis (22). As a result, we found that Caco-2 cells exposed to *C. albicans* in the presence of gliadin released higher levels of IL-15 when compared with each of the treatments used alone (Fig 1D). In vivo, WT mice fed a standard diet and infected with the fungus displayed increased intestinal IgA levels against gliadin (AGAb) and TG2 (Fig 1E), in line with previous reports showing increased anti-AGAb levels in subjects with chronic mucocutaneous candidiasis (23, 24). These results indicate that gliadin peptides have a direct impact on *C. albicans* morphotype likely contributing to CD pathogenesis.

### *C. albicans* contributes to the intestinal pathology in murine models of CD

**Figure S2.**
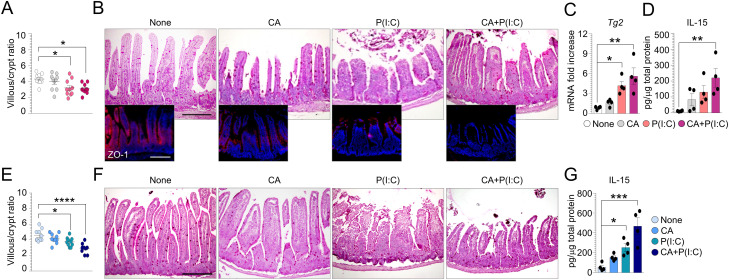
*Candida albicans* increases poly (I:C)–induced small-intestinal injury. **(A, B, C, D, E, F, G)** WT (A, B, C, D) and *Tph1*^*−/−*^ (E, F, G) mice were infected intragastrically with 1 × 10^9^ *Candida albicans* and treated intraperitoneally with 15 mg/kg poly (I:C) [P(I:C)] 12 h after infection. **(A, E)** Mice were evaluated 2 d after infection for (A, B, C, D, E, F, G) villous/crypt ratio (each data point corresponds to a single crypt/ratio evaluation in different high-power fields, n = 10), (B, F) histology (PAS staining) and ZO-1 immunofluorescence in small intestine, (C) *Tg2* expression by RT–PCR and (D, G) IL-15 production evaluated in small intestine by ELISA. Data are expressed as mean ± SEM from three independent experiments (n = 3–4 mice/group). Images were taken with a high-resolution microscope (Olympus BX51), 20× magnification (scale bars, 200 μm). *P < 0.05, ***P < 0.001, ****P < 0.0001. One-way ANOVA, Bonferroni multiple comparisons test. Treated versus untreated (None) mice. CA, *Candida albicans*. Source data are available for this figure.

It is well known that genomic dsRNA from rotavirus, and its synthetic analog [P(I:C)], induces severe mucosal injury in the small intestine and is associated with the onset of CD (25, 26, 27). We resorted to a mouse model of poly (I:C) [P(I:C)]–driven small intestine damage to evaluate the impact of *Candida* in the early phase of CD pathogenesis. WT mice were infected with *C. albicans* and/or treated intraperitoneally with 15 mg/kg [P(I:C)] 12 h after the infection. We found that mice treated with [P(I:C)] and infected with *C. albicans*, although not showing a worsening in the intestinal architecture (Fig S2A and B), displayed a reduced expression of the tight junction zonula occludens-1 (ZO-1) (Fig S2B), a key regulator of intestinal barrier function (28), and up-regulated *Tg2* expression (Fig S2C). These findings are in line with the observations that *C. albicans* is able to modulate epithelial tight junctions in a reversible manner by shifting ZO-1 from the membrane to cytoskeletal areas of the cell and to induce human and murine enterocytes death by enhancing TG2 activity (29). Importantly, mice infected with the fungus and treated with [P(I:C)] also released higher levels of IL-15 (Fig S2D), further indicating that *C. albicans* may contribute to the early stage of CD pathogenesis.

**Figure 2.**
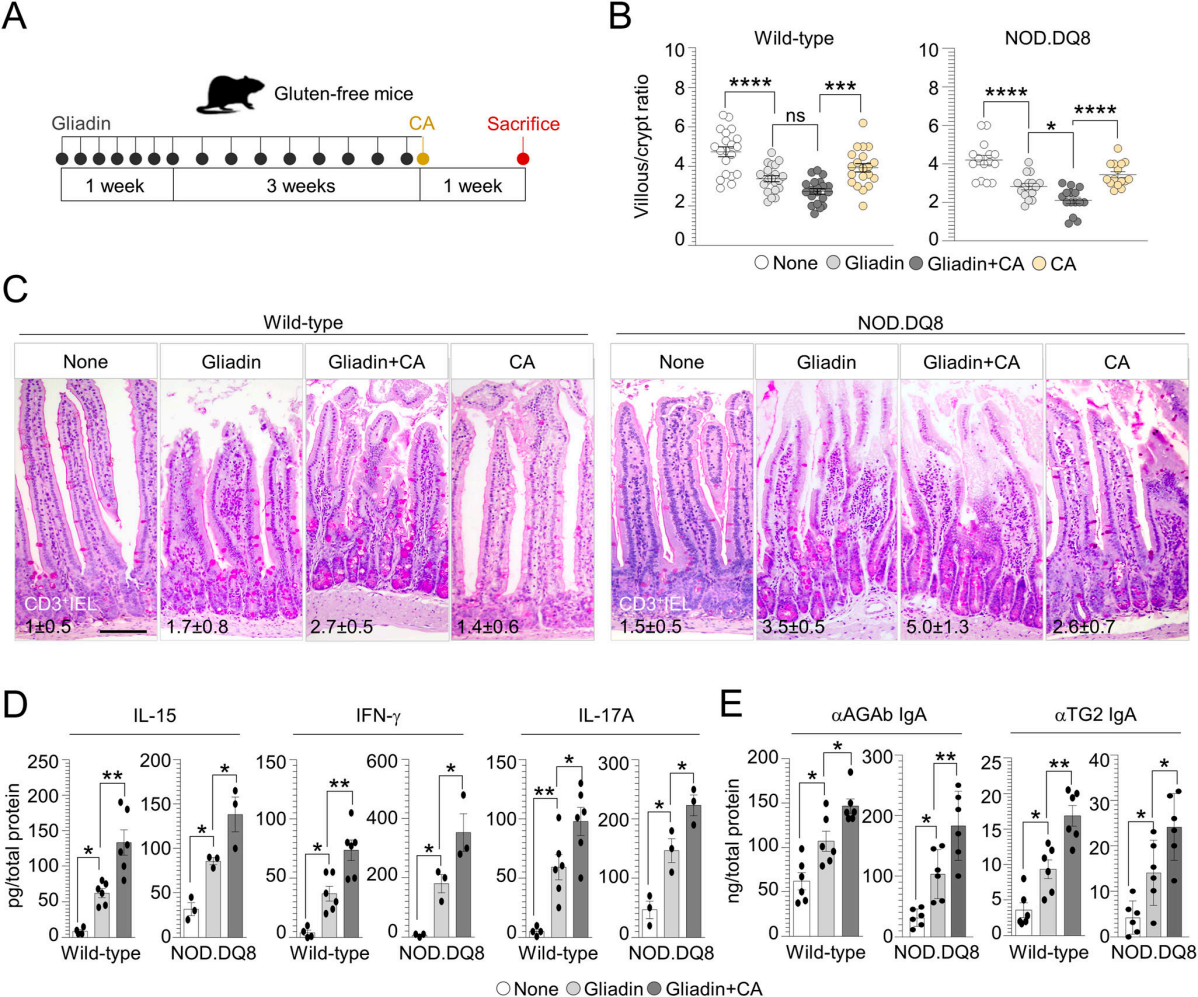
*Candida albicans* exacerbates intestinal pathology in gluten-sensitized mice. **(A)** WT and NOD.DQ8 mice were challenged via gavage with 500 mg/kg gliadin peptides and infected intragastrically with 1 × 10^9^ *Candida albicans* as depicted in the experimental schedule (A). **(B, C, D, E)** Mice were euthanized 7 d after infection and evaluated for (B) villous/crypt ratio (each data point corresponds to a single crypt/ratio evaluation in different high-power fields, n = 15–20), (C) small intestine histology (PAS staining) with relative number of CD3^+^ intraepithelial lymphocytes, (D) inflammatory cytokine production, and (E) anti(α)-gliadin (AGAb) and α-TG2 IgA production by ELISA in the small intestine of WT and NOD.DQ8 mice. Images were taken with high-resolution microscope (Olympus BX51), 20× magnification (scale bar 60 μm). Data are expressed as mean ± SEM from two to three independent experiments (n = 3–6 mice/group). *P < 0.05, **P < 0.01, ****P < 0.0001. One-way ANOVA, Tukey’s or Bonferroni multiple comparisons test. Gliadin-treated versus untreated mice (None), gliadin versus gliadin+CA–treated mice. CA, *Candida albicans*. Source data are available for this figure.

**Figure S3.**
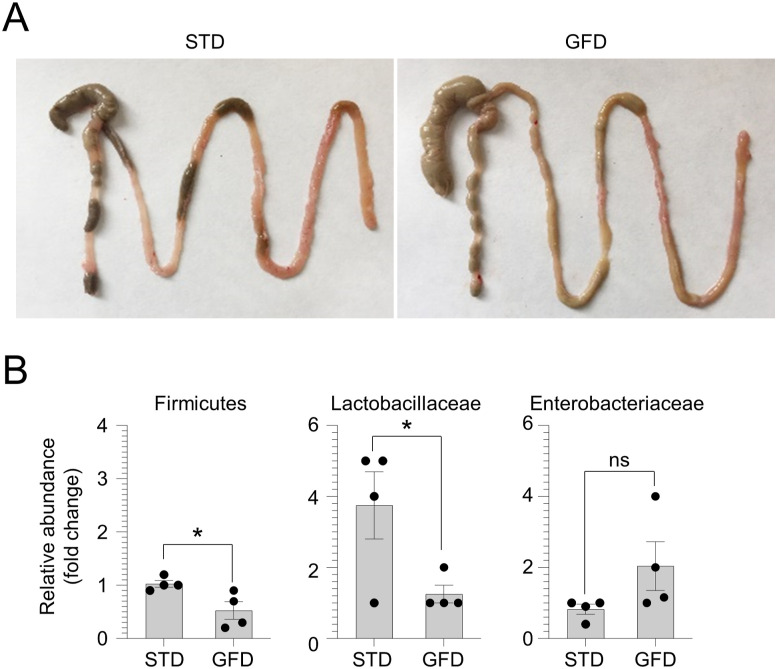
Evaluation of gluten-free diet (GFD) feeding. **(A, B)** WT mice were fed with GFD for at least three generations and evaluated for (A) intestinal gross evaluation and (B) relative abundance (fold change) of fecal bacteria. Data are expressed as mean ± SEM from three independent experiments (n = 4 mice/group). *P < 0.05, GFD versus standard diet. Unpaired *T* test. Source data are available for this figure.

**Figure S4.**
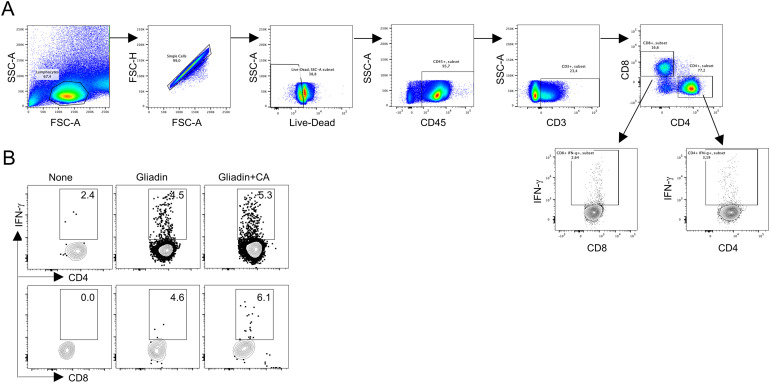
Flow cytometry analysis in gluten-sensitized NOD.DQ8 mice infected with *Candida albicans*. **(A, B)** Gating strategy for flow cytometry analysis and (B) CD4^+^ and CD8^+^IFN-γ^+^ T cells assessed in mesenteric lymph nodes of gluten-sensitized NOD.DQ8 mice infected with *C. albicans*. CA, *C. albicans*.

To validate these findings in well-established murine models of CD, WT mice inbred for at least three generations on a gluten-free diet (GFD), and nonobese diabetic (NOD) mice expressing the CD-predisposing HLA molecule DQ8 (NOD.DQ8), were subjected to gluten sensitization by gliadin challenge via gavage followed by *C. albicans* infection (Fig 2A). The gross examination of the intestine in untreated GFD mice revealed an increase in the dimension of the cecum (Fig S3A), likely due to a defective fermentation of dietary fibers and changes in microbiome composition (30). Indeed, feces from GFD mice showed a significant decrease in the abundance of Firmicutes and Lactobacillaceae, whereas Enterobacteriaceae were slightly increased (Fig S3B), in line with previous observations on the abundance of bacterial populations in healthy subjects on GFD (31). No structural alterations of small intestine and colon were observed in GFD mice compared with the control group (data not shown). Upon gluten sensitization, we found that gluten-sensitized mice infected with *C. albicans* showed altered intestinal architecture and inflammation (Fig 2B and C), with a lower villous-to-crypt ratio and higher CD3^+^ intraepithelial lymphocyte number than untreated and gliadin-only treated mice, and this was particularly evident in NOD.DQ8 mice (Fig 2B and C). This was accompanied by an increase in the number of CD4^+^ and CD8^+^ T cells producing IFN-γ in mesenteric lymph nodes (Fig S4B) and enhanced production of pro-inflammatory cytokines (Fig 2D) as well as mucosal anti-gliadin (AGAb) and TG2 IgA production (Fig 2E) in small intestine.

### The mast cells–*C. albicans* axis is involved in CD pathogenesis

**Figure 3.**
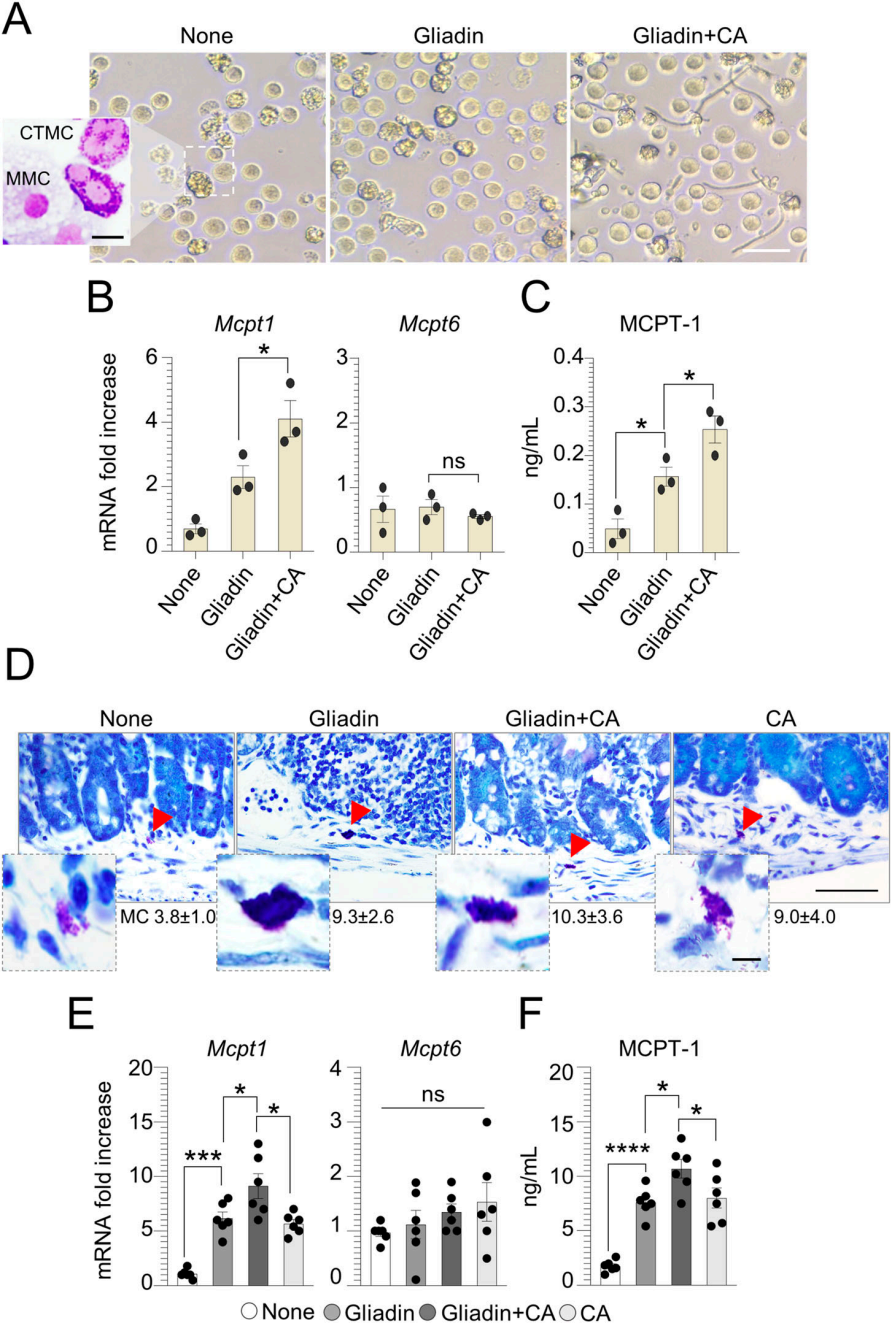
Mast cells–*Candida albicans* axis is involved in celiac disease. Bone marrow–derived mast cells from C57BL/6 mice were cultured with pepsin–trypsin–gliadin peptides (500 μg/ml) and pulsed with *Candida albicans*. **(A, B, C)** After 12 h of culturing, cells were evaluated for (A) morphology by light microscope and May–Grünwald Giemsa staining (on inset), (B) MC-related proteases expression by RT–PCR, and (C) MCPT-1 production by ELISA. In (D, E, F), WT mice were challenged via gavage with 500 mg/kg gliadin peptides and infected intragastrically with 1 × 10^9^ *Candida albicans*. **(D, E, F)** Mice were euthanized 7 d after infection and assessed for (D) MC evaluation by Toluidine blue staining with relative MC count, (E) MC proteases expression by RT–PCR, and (F) serum MCPT-1 by ELISA. Images were taken with high-resolution microscope (Olympus BX51), 100× magnification (scale bars 20 μm). Red arrows indicate mast cells. Data are expressed as mean ± SEM from two to three experiments (n = 4 mice/group). *P < 0.05, **P < 0.01, ****P < 0.0001. One-way ANOVA, Bonferroni multiple comparisons test. Gliadin versus untreated (None) cells or mice, gliadin+CA versus gliadin-treated cells or mice. CA, *Candida albicans*; MMC, mucosal mast cells; CTMC, connective-tissue mast cells; ns, not significant. Source data are available for this figure.

Although CD is considered a T cell–ediated enteropathy, MCs have been associated with the development of the disease (18). Given that the changes towards an inflammatory condition driven by MCs contribute to switch the *Candida* behavior from commensal to pathogen and exacerbate mucosal inflammation (20, 32), we looked for the contribution of the MCs-*Candida* axis to CD pathogenesis. We first stimulated bone marrow-derived mast cells (BMMCs) obtained from C57BL/6 mice with gliadin peptides in the presence of *C. albicans*. After 12 h of culture, although no relevant differences in MC morphology were observed (Fig 3A), we found that *C. albicans* amplified the ability of gliadin peptides to promote an inflammatory MC phenotype, as revealed by the increased levels of MCPT-1 (Fig 3B and C). It is well known that MCPT-1, a chymase massively released by mucosal MCs (MMCs), contributes to increase epithelial paracellular permeability during intestinal nematode infections (33, 34). On the contrary, the expression of *Mcpt6*, a tryptase produced by connective-tissue MCs (CTMCs) with an anti-inflammatory activity during intestinal candidiasis (20), was unaffected in MCs treated with gliadin peptides and pulsed with *C. albicans* (Fig 3B). To validate these results in vivo, we looked for MC expansion and function. We found that whereas *C. albicans* apparently did not promote the expansion of MCs in gluten-sensitized mice, as seen by toluidine blue staining (Fig 3D), the fungus was able to induce a MC inflammatory phenotype. Indeed, gluten-sensitized and infected mice showed an increase in MCPT-1 expression and production (Fig 3E and F) and decrease *Mcpt6* expression (Fig 3E), indicating that *C. albicans* may affect MC function in CD.

### The IL-9–mast cell axis is pathogenic in CD

**Figure 4.**
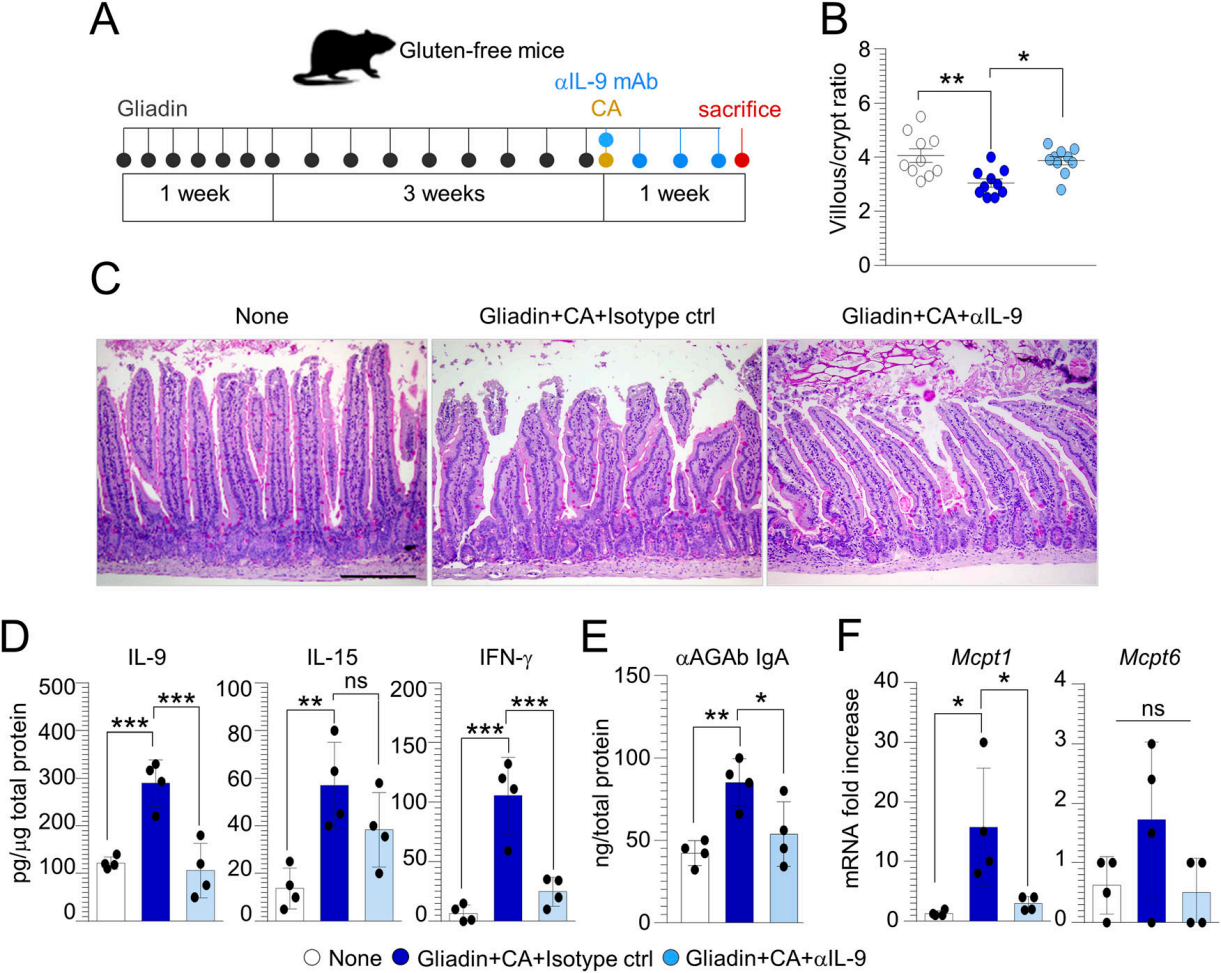
IL-9 neutralization ameliorates intestinal pathology in gluten-sensitized mice infected with *Candida albicans*. **(A)** WT mice were challenged via gavage with 500 mg/kg gliadin peptides, infected intragastrically with 1 × 10^9^ *Candida albicans* and treated with anti(α)–IL-9 or isotype control mAb as depicted in the experimental schedule (A). **(B, C, D, E, F)** Mice were evaluated for (B) villous/crypt ratio (each data point corresponds to a single crypt/ratio evaluation in different high-power fields, n = 10), (C) histology (PAS staining) of small intestine, (D) cytokine production, and (E) anti(α)-gliadin (AGAb) IgA production by ELISA and (F) MC proteases expression by RT–PCR in the small intestine. Data are expressed as mean ± SEM from three independent experiments (n = 3–4 mice/group). Images were taken with a high-resolution microscope (Olympus BX51), 20× magnification (scale bars, 200 μm). *P < 0.05, **P < 0.01, ***P < 0.001. One-way ANOVA, Bonferroni multiple comparisons test. Gliadin+CA+Isotype ctrl versus untreated (None) mice, gliadin+CA+αIL-9 versus gliadin+CA+Isotype ctrl mice. CA, *Candida albicans*; ns, not significant. Source data are available for this figure.

IL-9 is a pleiotropic cytokine that has been often associated with intestinal diseases, from food allergy (35) to inflammation (36). Besides innate lymphoid cells 2 and T helper 9 cells, IL-9 is mainly produced by a specific subset of MMCs on which IL-9 promotes survival and recruitment. Previously, we have shown the increased positivity of IL-9 in CD biopsies and IL-9 production in gluten-sensitized mice particularly upon *C. albicans* infection (20), suggesting that IL-9 could be an attractive drugable pathway in CD. To evaluate whether the IL-9 neutralization may ameliorate the intestinal pathology exacerbated by *C. albicans*, we treated gluten-sensitized and infected mice with anti–IL-9 or isotype mAb (Fig 4A). We found the amelioration of intestinal pathology in terms of increased villous/crypt ratio (Fig 4B) and intestinal architecture (Fig 4C), with a significant reduction of IL-9 and IFN-γ (Fig 4D) along with αAGAb production (Fig 4E). However, IL-9 neutralization failed to significantly decrease IL-15 production (Fig 4D), while reducing MC expression of *Mcpt1* (Fig 4F), these finding suggesting that IL-9 neutralization may be exploited, to some extent, to prevent intestinal pathology in CD.

### The gut microbiota composition and function are altered in CD and restored by a microbial metabolite

**Figure 5.**
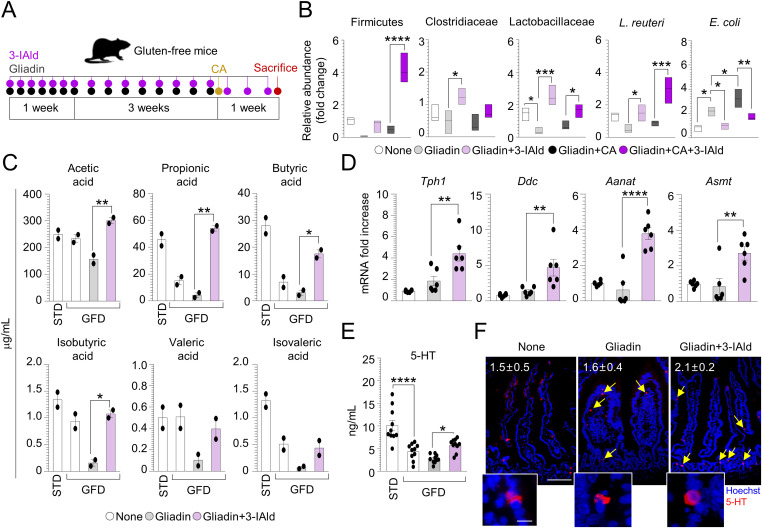
Indole-3-aldehyde modulates microbiota composition and metabolic profile in celiac disease. **(A)** WT mice challenged with gliadin peptides and infected intragastrically with *Candida albicans* were treated with indole-3-aldehyde (3-IAld) as depicted in the experimental schedule (A). **(B, C, D, E, F)** Mice were euthanized 7 d after infection and evaluated for (B) bacterial abundance in fecal samples, (C) short-chain fatty acids levels in fecal samples, (D) 5-HT-related genes expression by RT–PCR, (E) 5-HT production, and (F) positivity in the small intestine with relative quantification. Images were taken with high-resolution microscope (Olympus BX51), 20× and 40× magnification (scale bars 60 μm and 100 μm, respectively). Hoechst 33342 was used to counterstain nuclei. Data are expressed as mean ± SEM from three independent experiments (n = 3–6 mice/group). *P < 0.05, **P < 0.01, ***P < 0.001, ****P < 0.0001. One-way ANOVA, Bonferroni multiple comparisons test. Gliadin+3-IAld versus gliadin treated mice, gliadin+CA+3-IAld versus gliadin+CA treated mice, gluten-free diet versus standard diet. CA, *Candida albicans*; None, untreated mice. Source data are available for this figure.

The gut microbiome is an important contributing factor to the pathogenesis of CD, including a decrease expansion of *Lactobacillus* spp. and Bifidobacterium (37, 38), a microbial finding predicting an increased pathogenicity of *C. albicans* (39, 40). In line with these observations, we confirmed a decreased abundance of the Firmicutes phylum, Clostridiaceae, and Lactobacillaceae families and *Lactobacillus reuteri* in our CD mice (Fig 5B). This microbial composition resulted in an increase of *C. albicans* pathogenicity that led to a significant expansion of the intestinal pathogen *E. coli* in gluten-sensitized infected mice (Fig 5B). To evaluate whether the modulation of microbiota may have a protective activity in CD, we resorted to the microbial metabolite, indole-3-aldehyde (3-IAld) (Fig 5A), known to play a therapeutic role in mucosal homeostasis and microbial dysbiosis (41). It is known that patients with active CD have impaired capacity to metabolize tryptophan (trp), an amino acid and essential component of the human diet, and to produce microbial metabolites such as 3-IAld (42). We found that 3-IAld decreased the expansion of *E. coli*, whereas restored Firmicutes, Lactobacillaceae, Clostridiaceae as well as of *L. reuteri*, known to antagonize *C. albicans* infectivity in the gut (41) (Fig 5B). Consistently, 3-IAld significantly restored the fecal levels of short-chain fatty acids (SCFAs) strongly involved in the maintenance of gut function (43) (Fig 5C) and known to be defective in CD children (44, 45, 46).

**Figure S5.**
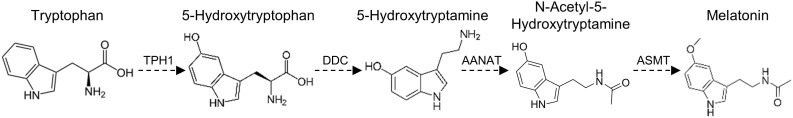
The biochemical pathway of serotonin synthesis and metabolism in the gut. TPH1, tryptophan hydroxylase 1; DDC, aromatic L-amino acid decarboxylase; AANAT, serotonin N-acetyltransferase; ASMT, N-acetylserotonin O-methyltransferase.

Among the trp catabolites, serotonin (5-HT) is an emerging key player in intestinal immune homeostasis and host microbiota cross-talk (47). Being 5-HT mainly produced within the gut by enterochromaffin cells through SCFAs (48), we looked for 5-HT production in gluten-sensitized mice treated with 3-IAld. To this purpose, we evaluated the expression of 5-HT–associated genes (Fig S5) and found that 3-IAld–treated mice showed an increase in the expression of *Tph1* and *Ddc* (Fig 5D), the rate-limiting enzymes crucial for 5-HT synthesis, and a significant up-regulation of *Aanat* and *Asmt* genes (Fig 5D) that catalyze the conversion of 5-HT to melatonin. Accordingly, 3-IAld increased 5-HT production (Fig 5E) and the number of 5-HT–positive cells in the gut of gluten-sensitized mice (Fig 5F). The protective effect of 5-HT observed in gluten-sensitized mice was corroborated also by the increased intestinal damage (Fig S2E and F) along with a significant release of IL-15 (Fig S2G) in the small intestine of *Tph1*^*–/–*^ mice subjected to [P(I:C)] model. These data suggest that 3-IAld modifies intestinal microbiota composition and function toward protective bacteria and metabolites, including 5-HT.

**Figure S6.**
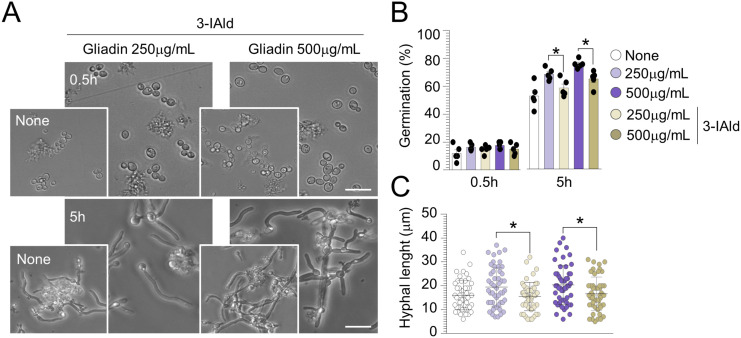
Indole-3-aldehyde counteracts the effects of gliadin peptides on *Candida albicans* morphology. *Candida albicans* (SC5314) yeasts were exposed at different concentrations of gliadin peptides and indole-3-aldehyde (3-IAld) in RPMI-1640 liquid medium. **(A, B, C)** Cells aliquots were imaged at different time points (A) by microscopy and evaluated for (B) cell germination and (C) hyphal length (a minimum of 50 hyphae were analyzed in different high-power fields). Images were taken with a high-resolution microscope (EVOS FL Auto Cell Imaging System), 100× magnification (scale bar, 20 μm). Data are expressed as mean ± SEM of two independent experiments. *P < 0.05. Two-way ANOVA, Tukey’s multiple comparisons test. Gliadin-treated+3-IAld versus gliadin-treated cells. None, untreated cells.

Of great interest, we found that 3-IAld inhibited the fungal germination and reduced the hyphal length exerted by gliadin peptides on *C. albicans* (Fig S6A–C), a finding confirming the ability of 3-IAld to control *Candida* morphogenesis (49) and pointing to the multitasking protective activity of 3-IAld at the host–pathogen interface.

### Indole-3-aldehyde–modified microbiota and metabolites prevents CD intestinal pathology

**Figure 6.**
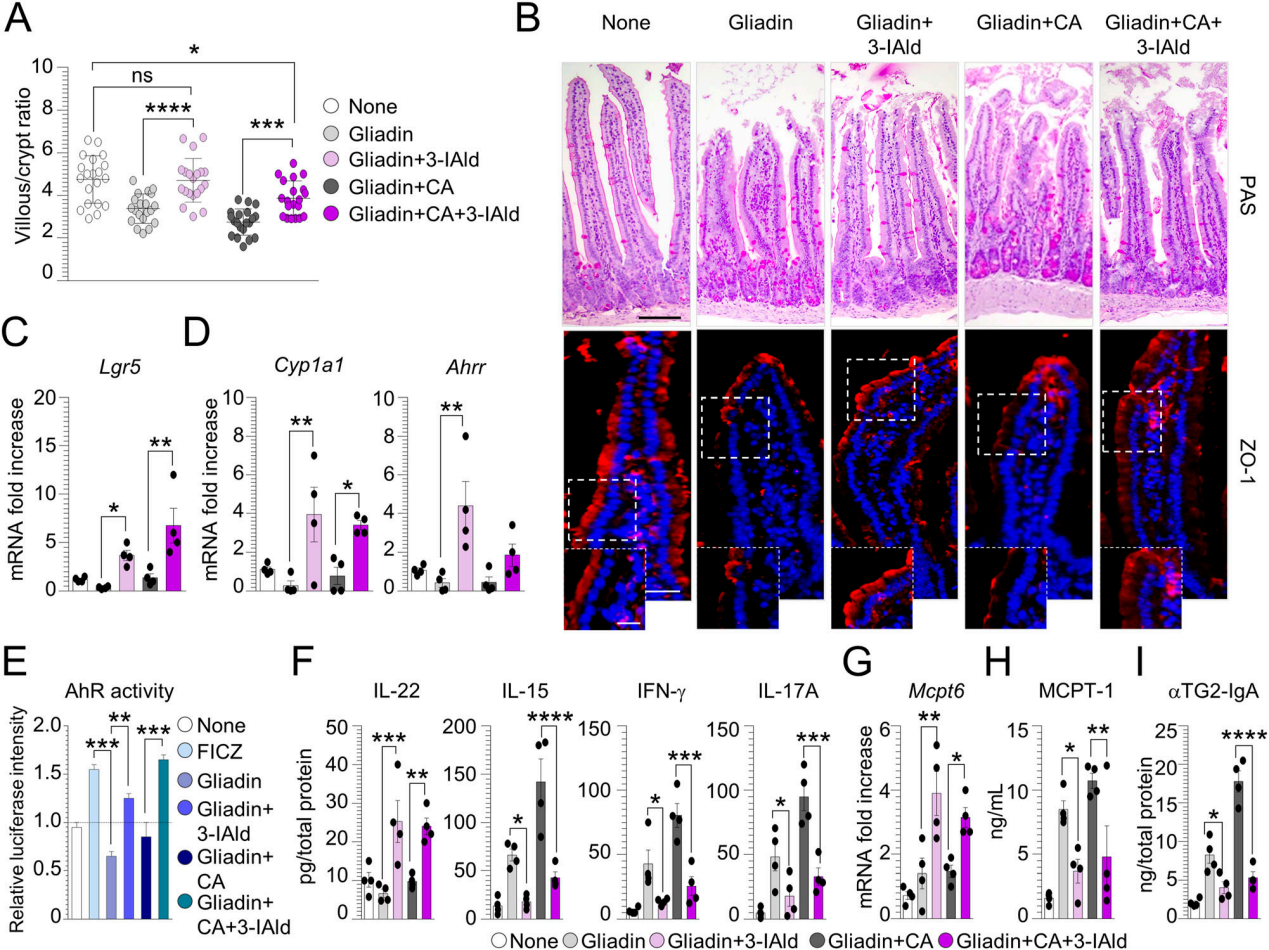
Indole-3-aldehyde protects from celiac disease intestinal pathology. WT mice were challenged via gavage with 500 mg/kg gliadin peptides, infected intragastrically with 1 x 10^9^ *Candida albicans* and treated with 18 mg/kg indole-3-aldehyde (3-IAld). **(A, B, C, D, E, F, G, H, I)** Mice were euthanized 7 d after infection and evaluated (A) for villous/crypt ratio (each data point corresponds to a single crypt/ratio evaluation in different high-power fields, n = 20), (B) small intestine histology (PAS staining) and zonula occludens-1 (ZO-1) immunofluorescence, (C) *Lgr5* and (D) AhR-related genes expression by RT–PCR, (E) AhR activity by luciferase assay [6-formylindolo(3,2-b)carbazole (FICZ) was used as a positive control], (F) cytokine production, (G) *Mcpt6* expression by RT–PCR in small intestine, (H) MCPT-1 production in serum, and (I) anti(α)-TG2 IgA production by ELISA. Images were taken with high-resolution microscope (Olympus BX51), 20 and 40× magnification (scale bars 60 μm and 100 μm, respectively). Hoechst 33342 was used to counterstain nuclei. Data are expressed as mean ± SEM from three independent experiments (n = 3–4 mice/group). *P < 0.05, **P < 0.01, ***P < 0.001, ****P < 0.0001. One-way ANOVA, Bonferroni multiple comparisons test. Gliadin+3-IAld– versus gliadin-treated mice, gliadin+CA+3-IAld– versus gliadin+CA–treated mice, gliadin- versus FICZ- or gliadin+3-IAld–treated cells, gliadin+CA+3-IAld– versus gliadin+CA–treated cells. None, untreated cells or untreated/uninfected mice; CA, *Candida albicans*; ns, not significant. Source data are available for this figure.

The restoration of microbial composition and metabolic profile upon 3-IAld treatment led us to evaluate whether its administration could ameliorate intestinal pathology in CD. We found that 3-IAld administration significantly ameliorated intestinal architecture (Fig 6A and B) and increased barrier function, both in uninfected and infected mice, as revealed by the restoration of the tight junction zonula occludens-1 (ZO-1) in the intestinal mucosal layer (Fig 6B). Similarly, the protective effect of 3-IAld was associated with the up-regulation of *Lgr5* (Fig 6C), an important proliferation marker associated with the intestinal regeneration and whose expression is maintained by *L. reuteri* (50). It has been indicated that *Lgr5*^*+*^ stem cells in the small intestine highly express the aryl hydrocarbon receptor (AhR) (51), a ligand-activated nuclear transcription factor able to orchestrate a protective immune response at mucosal barrier sites (52). Given that the reduced indoles levels in CD subjects led to a lower AhR activation that was efficiently restored in NOD.DQ8 mice by treatment with AhR agonists (53), we assessed AhR activation upon 3-IAld treatment. As expected, gluten-sensitized mice infected or not with *C. albicans* and treated with 3-IAld showed AhR activation, as revealed by the increased expression of AhR-related genes *Cyp1a1* e *Ahrr* (Fig 6D), the AhR-agonistic activity present in the small intestine (Fig 6E) and the restoration of the IL-22 levels (Fig 6F). This was paralleled by the reduction of IL-15, IFN-γ, and IL-17A, particularly in infected mice (Fig 6F). In addition, 3-IAld was able to regulate MC function shifting from inflammatory to protective MCs, as indicated by the increase expression of *Mcpt6* (Fig 6G) and decrease production of serum MCPT-1 (Fig 6H). Of interest, 3-IAld reduced intestinal anti-TG2 IgA in gluten-sensitized mice and even more upon *C. albicans* infection (Fig 6I). This result may be explained by the ability of 3-IAld to inhibit *Candida* germination, being Hwp1 an ideal substrate for TG2 cross-linking (54). Altogether, these results indicate that 3-IAld by maintaining the intestinal barrier integrity, restraining inflammation and controlling dysbiosis may represent an ideal therapeutic molecule in CD.

## Discussion

This study sheds light on the environmental factors influencing the commensal-to-pathogen switch of *C. albicans* in the gut that contribute to the intestinal pathology in CD. As primary reservoir of *C. albicans*, the gut is a perfect habitat where living as a harmless commensal and protecting the host from various microbial insults. However, inflammatory conditions perturbing this peaceful microenvironment have a relevant impact on *Candida* pathogenicity. MCs and IL-9 are able to finely tune *Candida* behavior in the gut by promoting commensalism or pathogenicity in a context-dependent manner (20). Herein, we demonstrated that the MC–IL-9–*Candida* axis is involved in CD pathogenesis and contributed to exacerbate the intestinal pathology. Although MCs are probably best known for their role in mediating IgE-associated allergic reactions (35), it has become increasingly clear that they comprise a heterogeneous cell population that can influence many pathophysiological processes. Indeed, their localization in the body and the capacity to react to different stimuli, releasing a huge type of mediators, point to MCs as important sentinels in host defense against bacterial, viral and parasitic infections (16). However, a perturbed milieu, such as in CD, promotes MCs to switch toward an inflammatory phenotype thus contributing to increase epithelial damage and intestinal permeability. This ability is potentiated by IL-9 that acts as amplifier on inflammatory MC function. The pathogenic effect of MC–IL-9 axis in CD could create a favorable condition for *Candida* commensal-to-pathogen transition and the consequent exacerbation of the intestinal pathology. We found that IL-9 neutralization and MC modulation counteracted the pathogenic potential of the IL-9–MC–*Candida* cross-talk eventually contributing to intestinal homeostasis. This result suggests that the IL-9–MC axis could be an attractive drugable pathway to prevent the clinically unwanted consequences of fungal colonization in CD. On this regard, MEDI−528, a humanized mAb against IL-9, is already used in clinics for the treatment of inflammation (55).

Gut microbiota and CD are intimately related. Changes in the intestinal microbiota composition and microbial-derived metabolites have been reported in CD patients and represent important risk factors (14, 56, 57). Therefore, the restoration of gut microbiome through microbial-based therapies might be useful for improving intestinal pathology in CD. Currently, the only available treatment for CD patients is a life-long GFD that is still associated with several challenges because of the high cost and the accidental exposure to gluten. Moreover, a subgroup of patients does not respond to the diet adequately, and healing of the small-bowel mucosa can be incomplete. Herein, we showed that the endogenous metabolite, 3-IAld, ameliorated the inflammatory pathology in murine CD through a primary action on gut mucosal and microbial homeostasis. Specifically, 3-IAld affected the microbiota composition leading to the expansion of protective taxa belonged to the Firmicutes phylum, like Clostridiaceae and Lactobacillaceae that have been found to be able to degrade and detoxify the immunogenic gliadin peptides (58). The ability of 3-IAld to provide protection through gut microbiota restoration has been already observed in murine models of immune checkpoint inhibitor-mediated colitis in which 3-IAld promoted the expansion of Lachnospiraceae and *Roseburia* spp. (59) that are, incidentally, reduced in CD subjects (60). It is known that *Roseburia* metabolizes dietary components producing high levels of butyrate, a four-carbon SCFA that enhances intestinal barrier function and mucosal immunity (61). Of interest, butyrate was shown to reduce gliadin-induced IFN-γ and IL-15 production from organoid-derived monolayers derived from CD biopsies (62), whereas IL-15 overexpression in the epithelium led to a reduction in butyrate-producing bacterial taxa, which increased susceptibility to colonic inflammation (63). Consequent to the gut microbiota restoration, 3-IAld was able to activate the host AhR/IL-22 axis likely contributing to the increased intestinal barrier function and decreased inflammation in gluten-sensitized mice. As a matter of fact, the beneficial effects of AhR restoration in improving gliadin-induced enteropathy upon specific probiotic treatment or dietary approaches has been already shown (53). One interesting observation of the present study was the ability of 3-IAld to modulate 5-HT production in the gut. Many evidences link CD to 5-HT. CD subjects show low levels of 5-HT in the brain that are restored after a strict GFD thus alleviating depressive and behavioral symptoms (64). By contrast, postprandial dyspepsia observed in CD patients is associated with the increased release of 5-HT, and this may account for the digestive symptoms (65). Of interest, 5-HT is covalently bound to small G-proteins in the cytoplasm by the endogenous TG2, a process named serotonylation that is involved in important cellular processes (66). Altogether, by promoting gut microbiota restoration, preventing intestinal damage, and regulating the host immune response, 3-IAld creates a favorable condition for the reinstatement of *Candida* commensalism in gluten-sensitized mice.

Sorting out the key factors that influence the commensal-to-pathogen switch of *C. albicans* in CD may be instrumental for pathogenesis and therapy. This study has deciphered some important variables underlying the intriguing role of *C. albicans* in CD. A better definition of the host microbiota–*C. albicans* cross-talk might provide a unique opportunity for developing novel therapeutics that working at the interface between microbes and the host immune system could be used in CD subjects to alleviate pathology and symptoms.

## Materials and Methods

### Ethics statement

The in vivo experiments were performed according to the Italian Approved Animal Welfare Authorization 874/2018-PR and Legislative decree 26/2014 regarding the animal licence obtained by the Italian Ministry of Health from 2018 to 2021.

### Peptic and trypsin-digested gliadin preparation

Pepsin–trypsin–gliadin (PT-gliadin) was obtained as follows. Briefly, 50 g Sigma-Aldrich gliadin (G-3375; Sigma-Aldrich) was dissolved in 500 ml 0.2 N HCl for 2 h at 37°C with 1 g pepsin (P-7000; 800–2,500 U/mg protein; Sigma-Aldrich). The peptic digest was further digested by addition of 1 g trypsin (P-8096, activity, 4× USP specifications; Sigma-Aldrich), after pH adjusted to 7.4 using 2 M NaOH. The solution was stirred vigorously at 37°C for 4 h, boiled to inactivate enzymes for 30 min, lyophilized, and then stored at −20°C until used. PT-gliadin was freshly suspended in a sterile phosphate-buffered saline.

### *Candida albicans* hyphal growth assay

*C. albicans* blastospores (SC5314) were seeded in RPMI-1640 liquid medium at 37°C and 5% CO_2_ for 5 h. Cells were exposed at different concentrations of gliadin peptides and 10 μM indole-3-aldehyde (3-IAld) and aliquots were imaged by microscopy. Under the microscope, selected germinated and ungerminated yeasts were counted in five different representative zones, randomly selected. The sum of germinated yeasts over total cells present determined the percentage of germination. Hyphal length was measured using NIH Image J software.

### Cells and treatments

Human colon adenocarcinoma–derived Caco-2 cells were maintained at 37°C in an atmosphere of 5% CO_2_/95% air and 90% relative humidity. EMEM medium (Euroclone) supplemented with 20% FSB, 1% nonessential amino acids, 1% L-glutamine, and 1% penicillin-streptomycin solution was used as culture medium. Cells were trypsinized and 1 x 10^6^ cells were seeded in a 24-well plate. After 24 h, cells were treated with different PT-gliadin concentrations and pulsed with *C. albicans* yeasts (ratio 1:1). BMMCs were obtained from 6- to 8- wk-old C57BL/6 mice by in vitro differentiation of bone marrow–derived progenitors obtained from mice femurs and tibiae. Precursor cells were cultivated in enriched medium (RPMI-1640 containing 2 mM L-glutamine, nonessential amino acids, 100 U/ml penicillin–streptomycin, 1 mM sodium pyruvate, 20 mM HEPES, 50 mM 2-mercaptoethanol, and 20% FCS) supplemented with 20 ng/ml murine rIL-3 alone and 100 ng/ml stem cell factor. After 8 wk of culture, BMMCs were monitored for FcεRI, c-Kit, and MHCII receptor expression by flow cytometry. Purity was usually more than 97%. 1 × 10^6^ differentiated MCs were stimulated with 500 μg/ml gliadin peptides and 10 μM 3-IAld and pulsed with *C. albicans* for 12 h. MCs were evaluated for morphology by light microscope and May-Grünwald Giemsa staining, and for MC proteases.

### Mice and treatments

WT C57BL/6 mice 8–10 wk old were purchased from Charles River Laboratories (Calco). Mice were inbred for at least three generations on a diet of gluten-free food pellets (Mucedola). NOD.DQ8 mice, which express HLA-DQ8 in an endogenous MHC class II-deficient background, were purchased from The Jackson Laboratory and maintained on a low-fat (4.4%) GFD (Mucedola srl). Mice were bred in a conventional specific pathogen-free colony at the San Raffaele Scientific Institute SOPF animal house (67). Gluten sensitivity was induced by challenging mice via gavage with 500 mg/kg gliadin every day for 1 wk and every other day for the following 3 wk. In concomitance to gluten sensitization, mice were treated intragastrically with 18 mg/kg 3-IAld, and then infected with 1 × 10^9^ *C. albicans*. Mice were euthanized 7 d after the infection. Poly(I:C) sodium (Sigma-Aldrich) was dissolved in pathogen-free saline and injected intraperitoneally (15 mg/kg).

### Enteric formulation preparation

Briefly, the enteric microparticles of 3-IAld were prepared using Eudragit L100 to S100 at a ratio of 1:2, with the addition of EC (30% w/w, ETHOCEL std. 7; Dow Chemical Company). 3-IAld (Sigma-Aldrich) and the polymers were dissolved in ethanol at a feedstock concentration of 3% wt/vol and spray-dried at an inlet temperature of 75°C using a Mini Spray-dryer model B-290 in the cocurrent mode, equipped with a two-fluid nozzle with a 0.7-mm nozzle tip and a 1.5-mm-diameter nozzle cap. The obtained dried microparticles were recovered by using a high-performance cyclone.

### Histology, immunofluorescence, and immunohistochemical staining

For histology, paraffin-embedded intestinal sections (3–4 μm) were stained with hematoxylin and eosin and evaluated for villus-to-crypt ratio. The villus height-to-crypt depth ratios were obtained from morphometric measurements of well-oriented villi (n = 4–12). The villus-to-crypt ratio was calculated by dividing the villus height by the corresponding crypt depth. The evaluation was performed using NIH Image J software. For immunofluorescence, intestinal sections were incubated at 4°C with anti-ZO-1 and 5-HT (Thermo Fisher Scientific) followed by secondary TRITC antibody (Sigma-Aldrich). Hoechst was used to detect nuclei. The villus-to-crypt ratio and cell fluorescence intensity was measured by using the ImageJ software. For immunohistochemistry staining, the intestinal sections were rehydrated and the antigens were retrieved by boiling in a citrate buffer (10 mM, pH 6). Subsequently, the endogenous peroxidase was quenched with 3% H_2_O_2_ for 10 min at RT and then incubated with a blocking buffer (10% Horse Serum in TBS). After rinsing, the slides were treated overnight at 4°C with primary antibody CD3 (Leica). The primary antibody was detected with a VECTASTAIN Elite ABC Kit (VECTOR Laboratories). Diaminobenzidine was used as a chromogen, followed by counterstaining with hematoxylin. For mast cells (MCs) count, sections were stained with Toluidine blue. The number of MCs was counted under a light microscope (40× magnification) and is expressed as cells per high power field. A minimum of four HFD was analyzed. Photographs were taken using a high-resolution BX51 microscope (Olympus) and captured using a high-resolution DP71 camera (Olympus).

### Flow cytometry analysis

Single-cell suspensions were obtained from mesenteric lymph nodes. The organs were minced and digested in Type IV Collagenase (3 mg/ml) and DNase I (50 mg/ml) in PBS + 5% FCS in a 37°C shaking water bath. After 45 min, digestion was stopped by adding cold PBS + 5 mM EDTA and single-cell suspensions were filtered through a 70-μm cell strainer and washed twice. For intracellular cytokine staining, cells were stimulated with PMA (50 ng/ml), ionomycin (2.5 μg/ml), and Brefeldin A (1 μg/ml) for 3 h in a 37°C, 5% CO_2_ incubator. Cells were then stained with 7-AAD Viability Dye (Beckman Coulter) and incubated with 30% BSA and rat anti-mouse CD16/32 (eBioscience) for 10 min at 4°C to block Fc receptors. Subsequently cells were surface-stained with APC-Cy7–conjugated CD45 (clone 30-F11; BioLegend), BV785-conjugated CD3 (clone 17A2; BioLegend), BV510-conjugated CD4 (clone RM4-5; BioLegend), and Alexa Fluor 700–conjugated CD8a (clone 53-6.7; BioLegend) for 45 min at 4°C. Cells where then fixed, permeabilized using saponin (Sigma-Aldrich), stained for IFN-γ (PE, clone XMG1.2; eBioscience), acquired on BD LSRFortessa, and analyzed with FlowJo 10.8.2 software. Gating strategy was provided in Fig S4A.

### ELISA and quantification of SCFAs

The levels of cytokines in the intestinal homogenates, sera, and culture supernatants were determined by specific ELISAs (R&D System) following the manufacturer’s instructions. Anti-gliadin and anti-TG2-IgA were evaluated by specific ELISAs (MyBioSource). The concentration of secreted cytokines was normalized to the total tissue protein and expressed as the picogram of cytokine per microgram of total protein. To measure SCFAs, 150 mg of feces was aseptically mixed with 1 ml sterile PBS buffer in a sterile tube and vortexed until uniformly suspended. The suspension was centrifuged at 12,000*g* for 10 min and the supernatant was filtered through a 0.45-μm membrane filter. The mice diluted plasma samples and supernatants of feces were analyzed by gas chromatography-mass spectrometry (GC-MS) using a 6890 GC and a 5973 A MSD (Agilent Technologies) with electron ionization (EI) source. A J&W carbowax GC column (Agilent Technologies) was used, whereas head space-Solid Phase MicroExtraction (HS-SPME), using a PDMS/CAR/DVB 2-cm fiber (Supelco, Sigma-Aldrich Merck), was used to extract (30 min fiber exposure at 65°C, under magnetic stirrer agitation) and transfer the analytes to the GC injection port. Before SPME sampling, 10% perchloric acid (10 μl) and a 10 μl volume of an acid acqueous solution of deuterium-labeled internal standards (IS) were added for HS extraction and then quantitation. A linear calibration curve was recorded in PBS, with scalar amounts of the chemical standards of SCFAs and a constant amount of deuterated internal standard solution, the same added to the actual samples, and processed as for the plasma samples. The peak area ratio (PAR) for the specific ions of each analyte and its corresponding deuterated IS was measured to construct the calibration curve for each short-chain acid and calculate the sample concentration.

### Real-time PCR

Real-time RT PCR was performed using CFX96 Touch Real-Time PCR Detection System and SYBR Green chemistry (Bio-Rad). Cells were lysed and total RNA was reverse transcribed with cDNA Synthesis Kit (Bio-Rad), according to the manufacturer’s instructions. The PCR primers sequences (5′-3′) were as follows:

β-*actin*: AGCCATGTACGTAGCCATCC and CTCTCAGCTGTGGTGGTGAA;

*Mcpt1*: TCGAAAAACAAATCATTCACAAA and GACCAGGCAAGGGAATTACA;

*Mcpt6*: TTCTGCGGAGGTTCTCTCAT and TACTGCTCACGAAGCTGCAC;

*Il15*: ACCAGCCTACAGGAGGCC and TGAGCAGCAGGTGGAGGTA;

*Lgr5*: CACCTCCTACCTAGACCTCAGT and GACCTCCTCAATGCAGAACGC;

*Cyp1a1*: ACAGTGATTGGCAGAGATCG and GAAGGGGACGAAGGATGAAT;

*Ahrr*: AGAGGGTTCCCCGTGCAG and ACTCACCACCAGAGCGAAGC;

*Tph1*: TCAGCCGAGAACAGTTGAATG and GTCTTTGAAGCCAGGGTGGT;

*Ddc*: GAGCTGGACAATCCCGACAA and GATCAGGGGCCGAAGATAGC;

*Aanat*: AGGAGTCTCAGCTTCTCCTAGT and CCTGTGTAGTGTCAGCGACT;

*Asmt*: TTCGACCTCTCGCCCTTCAG and GAACACGGTGACCTCGCTG.

### Evaluation of AhR activation

To assess the activation of AhR, we used mouse hepatoma cells (H1L6.1c3), kindly provided by Allison K. Ehrlich (Meyer Hall, University of California, Davis, United States), containing the stably integrated AhR xenobiotic responsive element driven by a firefly luciferase reporter plasmid, pGudLuc6.1 (68). Cells were maintained in MEMA (Gibco), supplemented with 10% fetal serum bovine, and 1% penicillin–streptomycin solution. Briefly, cells were plated in a 24-well plate and maintained at 37°C for 24 h. Then, cells were stimulated with gut homogenates of treated and infected mice for 24 h. 6-Formylindolo (3,2-b)carbazole was used as a positive control. After incubation, cells were washed with PBS and 100 μl of 1X lysis buffer (Tris phosphate 1 M, pH 7.8, EDTA 0.5 M, glycerol 50%, Triton X, and H_2_O) was added to each well. The plate was placed on the plate shaker at RT until cells were lysed. Luciferase activity was measured using 100 μl of 1X reaction buffer (Reaction Buffer 2X, luciferin 10 mM, ATP 100 mM, CoA 10 mM, and H_2_O) for 30 μl of cell lysate. Luciferase activity, normalized to sample protein concentration, was calculated as relative light units per microgram of protein and expressed as fold induction.

### Bacterial DNA extraction and quantitative PCR for microbiota analysis

Bacterial DNA from feces of littermates WT mice was extracted using a QIAamp DNA Stool Mini Kit (QIAGEN). Bacteria species-specific PCR was carried out with primers targeted on the 16S rRNA (69) using CFX96 Touch Real-Time PCR Detection System and SYBR Green chemistry (Bio-Rad). The amplification program was 94°C for 5 min, followed by 35 cycles of 94°C for 30 s, 30 s at the appropriate annealing temperature and 72°C for 30 s. A cycle of 72°C for 10 min concluded the program. Amplification products were detected by agarose gel electrophoresis on 1.8% agarose gel, Gel Red (Biotium) staining and UV transillumination. Bacterial abundances were expressed as relative 16S rRNA gene levels. The PCR primers sequences (5′-3′) were as follows:

Eubacteria: ACTCCTACGGGAGGCAGCAG and ATTACCGCGGCTGCTGG;

Firmicutes: GGAGYATGTGGTTTAATTCGAAGCA and AGCTGACGACAACCATGCAC;

Lactobacillaceae: TGGATGCCTTGGCACTAGGA and AAATCTCCGGATCAAAGCTTACTTAT;

Clostridiaceae: AGCGTTGTCCGGATTTACTG and CGCTTACCTCTCCGACACTC;

Enterobacteriaceae: CAGGTCGTCACGGTAACAAG and GTGGTTCAGTTTCAGCATGTAC;

*L. reuteri*: TGAAGAAGGTCTTCGGATCG and TAAATCCGGATAACGCTTGC;

*E. coli*: CAAGTCATCATGGCCCTTAC and CGGACTACGACGCACTTTAT.

### Statistical analysis

GraphPad Prism software 6.01 (GraphPad) was used for analysis. Data are expressed as means ± SEM. Horizontal bars indicate the means. Statistical significance was calculated by one- or two-way ANOVA (Tukey’s or Bonferroni’s post hoc test) for multiple comparisons and by two-tailed *t* test for single comparisons. The variance was similar in the groups being compared. We considered all *P*-values of 0.05 or less to be significant. The in vivo groups consisted of 3–6 mice/groups. The data reported are either representative of at least two-three experiments (histology and immunofluorescence) or pooled otherwise.

## Supplementary Material

Reviewer comments
